# Supplementary material for: Targeting bivalency de-represses Indian Hedgehog and inhibits self-renewal of colorectal cancer-initiating cells
Source: Nat Commun. 2019 Mar 29;10:1436. doi: 10.1038/s41467-019-09309-4 (PMC6441108; doi:10.1038/s41467-019-09309-4)
Supplement: Supplementary file 1 — Supplementary Information [file 41467_2019_9309_MOESM1_ESM.pdf]

Supplementary Information

Targeting bivalency de-represses Indian Hedgehog and inhibits self-renewal of colorectal cancer-initiating cells

Lima-Fernandes et al.

Probe	Target	[μM]	p value
UNC1999	EZH2	5	****
JQ1	BRD2/3/4/T	1	***
NI-57	BRPF1/2/3	10	**
A196	SUV420H1/2	10	*
Bay598	SMYD2	10	*
GSK591	PRMT5	10	*
SGC707	PRMT3	10	ns
PFI2	SETD7	10	ns
NVS-1	CECR2	1	ns
GSK484	PAD4	10	ns
OICR9429	WDR5	5	ns
PFI3	SMARCA2/4/PB1	10	ns
A366	G9a	5	ns
SGC0946	DOT1L	5	ns
GSK LSD1	LSD1	1	ns
Baz2-ICR	BAZ2A/B	5	ns
MS049	PRMT4/6	10	ns
MS023	PRMT type I	10	ns
LP99	BRD7/9	10	ns
PFI4	BRPF1B	10	ns
UNC1215	L3MBTL3	5	ns
SGCBP30	CREBBP/EP300	3	ns

Supplementary Table 1. List of all the chemical probes used in the study. Chemical probes, their respective target names, the concentrations used for the spheroid viability screen in Fig.1a, and the statistical significance calculated using one-way ANOVA. The same probe concentration was used for the organoid drug screen in Fig.1b. (n=4, one-way ANOVA) *$P<0.05$, ** $P<0.01$, ***$P<0.001$, ****$P<0.0001$.

	Origin	Stage	Mutations
LS174T	Primary Colon	II	KRAS, β-Catenin
POP92	Primary Colon	IV	APC, P53, BRAF
POP181	Lung Metastasis	IV	APC, P53, NRAS
POP66	Liver Metastasis	IV	BRAF, P53
POP164	Primary Colon	III	PIK3CA
CSC171C	Primary Colon	IV	APC, P53
CSC171L	Liver Metastasis	IV	APC, NRAS

Supplementary Table 2. List of all the cell models used in the study. The tissue of origin, stage of disease as well as major mutations are recapitulated.

UNC1999-upregulated Genes with H3K27me3 marked promoters:								
ARHGEF16	ONECUT3	ADAMTSL4	DPM3	HS1BP3	SEMA3G	PLXND1	MXD4	SLC9A3
GNB2	THEM6	RHPN1	FSTL3	CYC1	FBXW5	ABCA2	C9orf142	DPP7
CRTAC1	COMTD1	PKP3	CCDC86	PIGQ	METRN	APRT	PPP1R1B	GRN
TRIM28	C20orf27	ENTPD6	PPDPF	STMN3	PFKL	SLC25A1	INPP5J	TTLL12
NKD2	RGS14	MAFK	NSMF	GJB1	CTAG2	GAA		

UNC1999-upregulated Genes with bivalently marked promoters:								
SDC3	IHH	GPC1	FSCN1	HEY1	LY6E	GRINA	OPLAH	ENTPD2
DEGS2	CKB	SSTR5	HID1	METRNL	C2CD4C	REEP6	HELZ2	TMEM151A
PWWP2B	UNC5B							

UNC1999-upregulated Genes with H3K4me3 marked promoters:								
AURKAIP1	TNFRSF14	AGTRAP	CMPK1	IGSF9	TSEN15	SRP9	NOC2L	AGRN
SDF4	DVL1	VWA1	ATAD3A	ARHGEF16	ECE1	RAP1GAP	LYPLA2	SFN
SERINC2	TMEM54	CITED4	HPDL	ADAMTSL4	DPM3	IGSF8	ASPM	PHLDA3
LBR	HIST3H2A	HS1BP3	KCNF1	IGFBP2	CHPF	CCL20	GPC1	RNPEPL1
STT3B	POMGNT2	ECT2	TBL1XR1	GPX1	RPL29	NAA50	PODXL2	PLXND1
WFS1	EREG	TMEM129	MXD4	H2AFZ	MAD2L1	NKD2	SUB1	TNPO1
RGS14	DBN1	PPARD	CD2AP	DEK	KIAA1586	MARCKS	SF3B5	MAFK
ZNF92	CLDN3	POR	HSPB1	LAMTOR4	GNB2	IMPDH1	EPHB6	TMUB1
ADAM9	TCEA1	CPSF1	CLDN23	LYPLA1	GGH	ARMC1	HEY1	THEM6
PYCRL	TSTA3	MROH6	SCRIB	PLEC	GRINA	GPAA1	OPLAH	EXOSC4
DGAT1	SLC52A2	HSF1	SLC39A4	VPS28	MFSD3	PTBP3	ST6GALNAC4	HDHD3
C9orf16	PTGES2	ZDHHC12	FIBCD1	CACFD1	SLC2A6	AGPAT2	EDF1	FBXW5
NPDC1	ABCA2	C9orf142	DPP7	SSNA1	NSMF	MRPL41	PHF6	TIMP1
HPRT1	FLNA	FAM3A	SLC10A3	G6PD	CTAG2	PWWP2B	KIF11	HELLS
UBE2D1	CDK1	COMTD1	BUB3	FUOM	TMEM151A	UNC93B1	RNH1	PKP3
ANO9	RASSF7	CDHR5	SLC25A22	RPLP2	TSSC4	PHLDA2	SMPD1	KCNJ11
CCDC86	B3GAT3	PRDX5	EHD1	ZNHIT2	MAP3K11	CCDC85B	PITPNM1	GSTP1
DHCR7	TSKU	OAF	FAM109A	SCARB1	RAD51AP1	H2AFJ	TWF1	ACADS
CDX2	MIS18BP1	GMFB	NAA30	TRMT61A	INF2	ZBTB42	CEP170B	CRIP2
CHAC1	NUSAP1	RPLP1	RCN2	MMP15	TMEM8A	MRPL28	PIGQ	RHOT2
ZNF598	SLC9A3R2	PRSS33	UBALD1	ALDOA	PRSS8	C16orf58	ZNF267	TPPP3
SLC7A5	MVD	APRT	SUZ12	SEPT9	CLUH	SPNS2	SREBF1	TOP2A
G6PC3	GRN	SLC25A39	GPRC5C	CASKIN2	TRIM47	WBP2	LGALS3BP	GAA
ARHGDIA	DUS1L	FASN	SECTM1	ELOF1	ACTN4	CLPTM1	NUCB1	C2CD4C
POLRMT	MISP	FSTL3	TMEM259	ATP5D	C19orf24	REEP6	ONECUT3	ABHD17A
BTBD2	GNA11	NCLN	SH3GL1	ALKBH7	CAMSAP3	PRKCSH	GADD45GIP1	CCDC124
FKBP8	TMEM161A	LSR	RABAC1	CIC	GLTSCR2	SCAF1	EPN1	TRIM28
NTSR1	C20orf27	CENPB	CST3	ENTPD6	SCAND1	OGFR	SLC17A9	ARFGAP1
HELZ2	STMN3	RRP1	PFKL	COL6A1	PLXNB2	SLC25A1	COMT	ZDHHC8
ISG15	SDC3	CDK18	BOK	PPP1R2	FGFR4	ZNF680	CHPF2	RHPN1
CYC1	RPL35	ENTPD2	WDR13	ARL5B	PTDSS2	SLC39A13	NUDT8	ORAI1
C15orf39	METRN	BCAR1	KRT19	SLC38A10	BSG	SCAMP4	GDF15	ABHD12
PPDPF	INPP5J							

Supplementary Table 3. Differentially expressed genes in UNC1999 treated samples with H3K4me3, H3K27me3 or bivalently marked promoters. Annotation of H3K27me3, H3K4me3 or bivalent promoter marks within UNC1999-upregulated genes.

Supplementary Table 4. RT-qPCR primers used in this study.

	Forward	Reverse
CDX2	TGTGCGAGTGGATGCGGAA	CTTTCGTCCTGGTTTTCACTTGG
DHH	AGGATGAGGAGAACAGTGGAGC	TCAGTCACTCGTAGGCGCACTC
ECT2	GCAGTCAGCAAGGTGGCAAGTT	CTCTGGTGCAAGGATAGGTCCA
FABP2	GCTGCAAGCTTCCTTTTCAC	CTGAAATCATGGCGTTTGAC
GLI1	TGGTGTGGAAATGACTGGCA	AGTGGTACCGGTGTGGGA
IHH	GACCGCGACCGCAATAAGTA	GCCGAGTGCTCGGACTTGA
HEY1	GGAGCAAGGATCTGCTAAGCTA	GATAACGCGCAACTTCTGCC
BMP4	CTGGTCTTGAGTATCCTGAGCG	TCACCTCGTTCTCAGGGATGCT
KLF4	CCCACATGAAGCGACTTCCC	CAGGTCCAGGAGATCGTTGAA
NANOG	AATACCTCAGCCTCCAGCAGATG	TGCGTCACACCATTGCTATTCTTC
OCT4	GAGAACCGAGTGAGAGGCAACC	CATAGTCGCTGCTTGATCGCTTG
SHH	CCGAGCGATTTAAGGAACTCACC	AGCGTTCAACTTGTCCTTACACC
Axin2	CTCCTTATCGTGTGGGCAGT	CTTCATCCTCTCGGATCTGC
CD44	TCCAACACCTCCCAGTATGACA	GGCAGGTCTGTGACTGATGTACA
EPHB2	CGCCATCTATGTCTTCCAGGTG	GATGAGTGGCAACTTCTCCTGG
TBP	GGGCATTATTTGTGCACTGAGA	TAGCAGCACGGTATGAGCAACT
18S	AACCCGTTGAACCCCATT	CCATCCAATCGGTAGTAGCG
IHH (ChIP)	CATTCATACCAAGCCCCCGT	AGAATACCGAGGAACCCCGA
MYT1 (ChIP)	GGGTACCAGAACAGGAAGCA	TCTGTGAGCTCATCCAGTGC
Actin (ChIP)	CGACTTCTAAGTGGCCGCAA	CAGAGCAACTGCCCTGAAAG

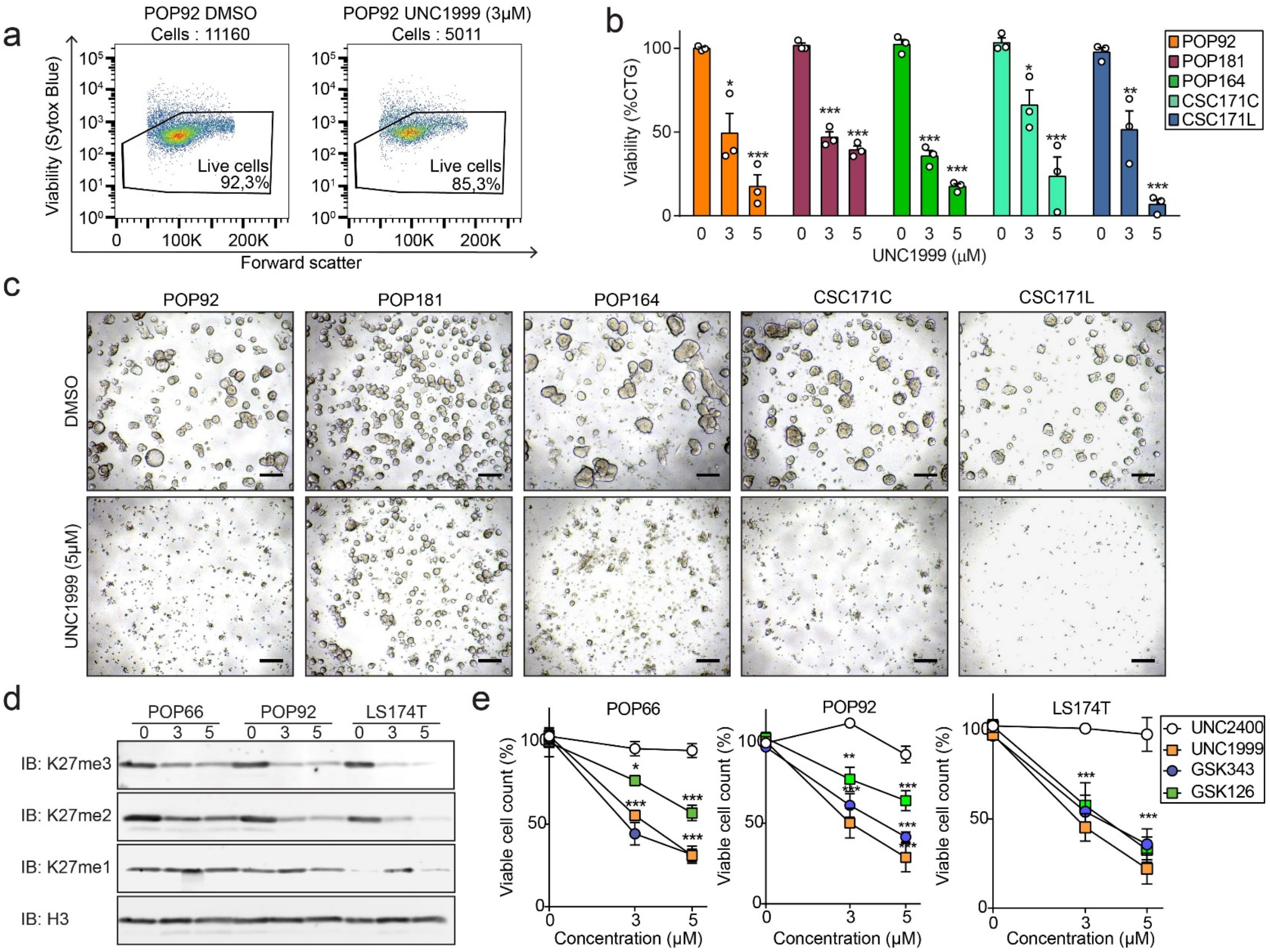

Supplementary Figure 1. EZH2 inhibition suppresses growth in patient-derived 3D colon cancer models. (**a**) Representative flow plots of POP92 viable cell counting assay. Cells were treated with UNC1999, processed for flow cytometry and stained with Sytox Blue viability dye. (**b-c**) Validation of the efficacy of UNC1999 at 3 and 5μM at suppressing growth in five PDO models using CelltiterGlo3D as readout. Data shown are mean of *n*=3 biological replicates +/- SEM, two-way ANOVA. Representative pictures of the PDOs 7 days after treatment are shown in (**c**). Scale bar is 100μm. (**d**) Representative western blot showing the reduction of H3K27me3 and H3K27me2 in three spheroid lines. The experiment was repeated *n*=3 times and representative images are shown. (**e**) Selective EZH2 inhibitors GSK343 and GSK126 were assessed for their effect on spheroid growth. UNC2400 was included as negative control. Data is represented as mean of *n*=4 independent experiments +/- SEM, two-way ANOVA. *P<0.05, ** P<0.01, ***P<0.001.

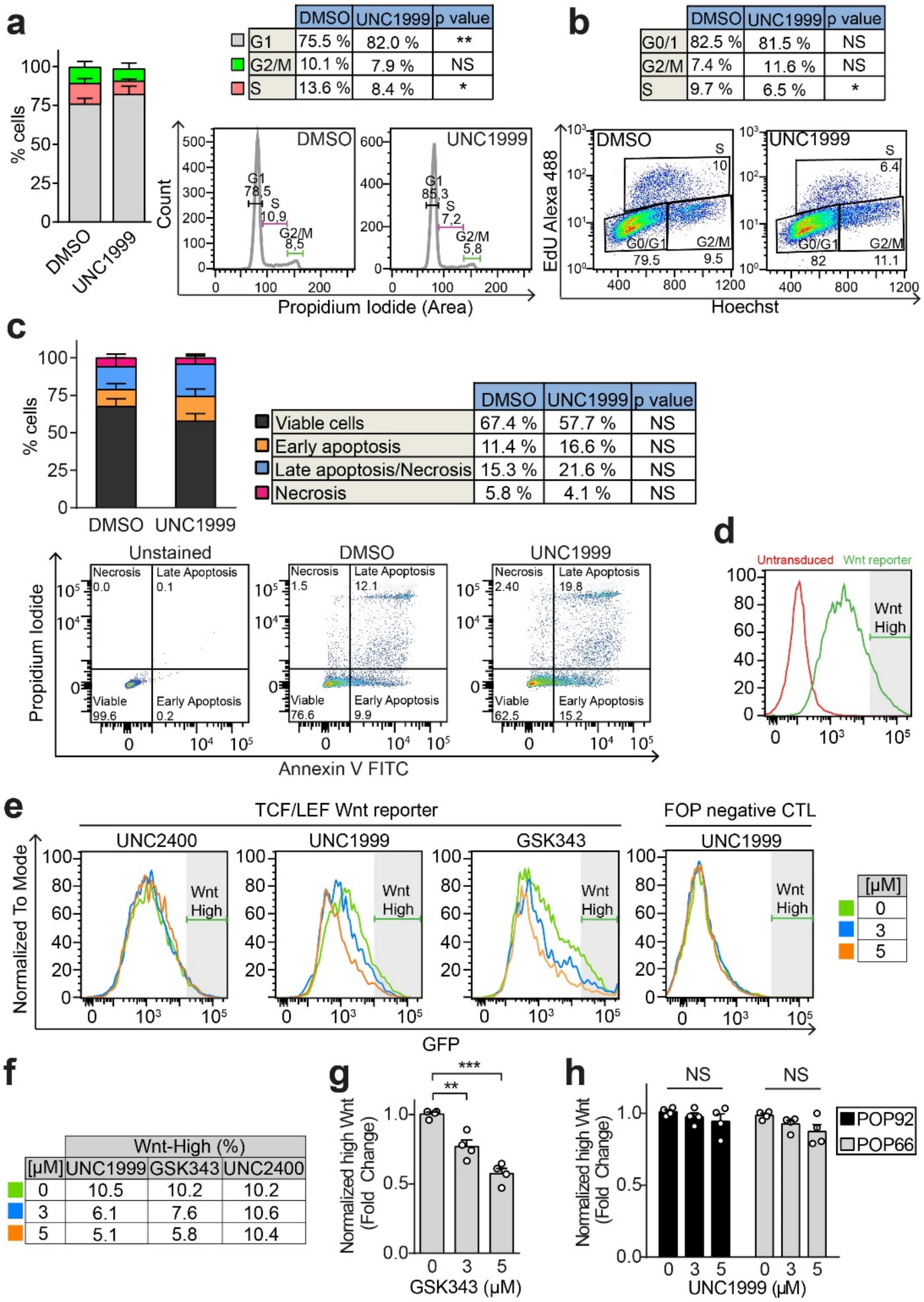

Supplementary Figure 2. Effect of EZH2 inhibition on cell cycle and apoptosis. (**a-b**) Cell cycle analysis (PI, (a), EdU/Hoechst (b)) of POP92 spheres treated with 3µM UNC1999 for 7 days. Data shown are *n*=8 (PI) or n=5 (EdU/Hoechst) +/- SEM, two-way ANOVA. Representative flow plots are shown. (**c**) Apoptosis/Necrosis assay (Annexin V/PI) performed on POP92 spheres treated with 3µM of UNC1999 for 7 days. Data shown are *n*=4 independent experiments +/- SEM, two-way ANOVA. Representative flow plots are represented in the bottom panels. (**d**) Representative flow cytometry histograms of POP92 CC-ICs stably expressing FOP mutated TCF/LEF-GFP reporter control and untransduced cells. (**e-h**) POP92 TCF/LEF GFP reporter cells or FOP negative control cells, treated with 3µM UNC1999, UNC2400 or GSK343 for 7 days and processed for Flow Cytometry. Quantification shown in (**f-h**) is performed on gating in the 10% Wnt-High in DMSO control as shown in (**e**). Data shown is mean of n=4 (f-h), +/- SEM, Student's t test (**g**), two-way ANOVA (**h**). *P<0.05, ** P<0.01, ***P<0.001.

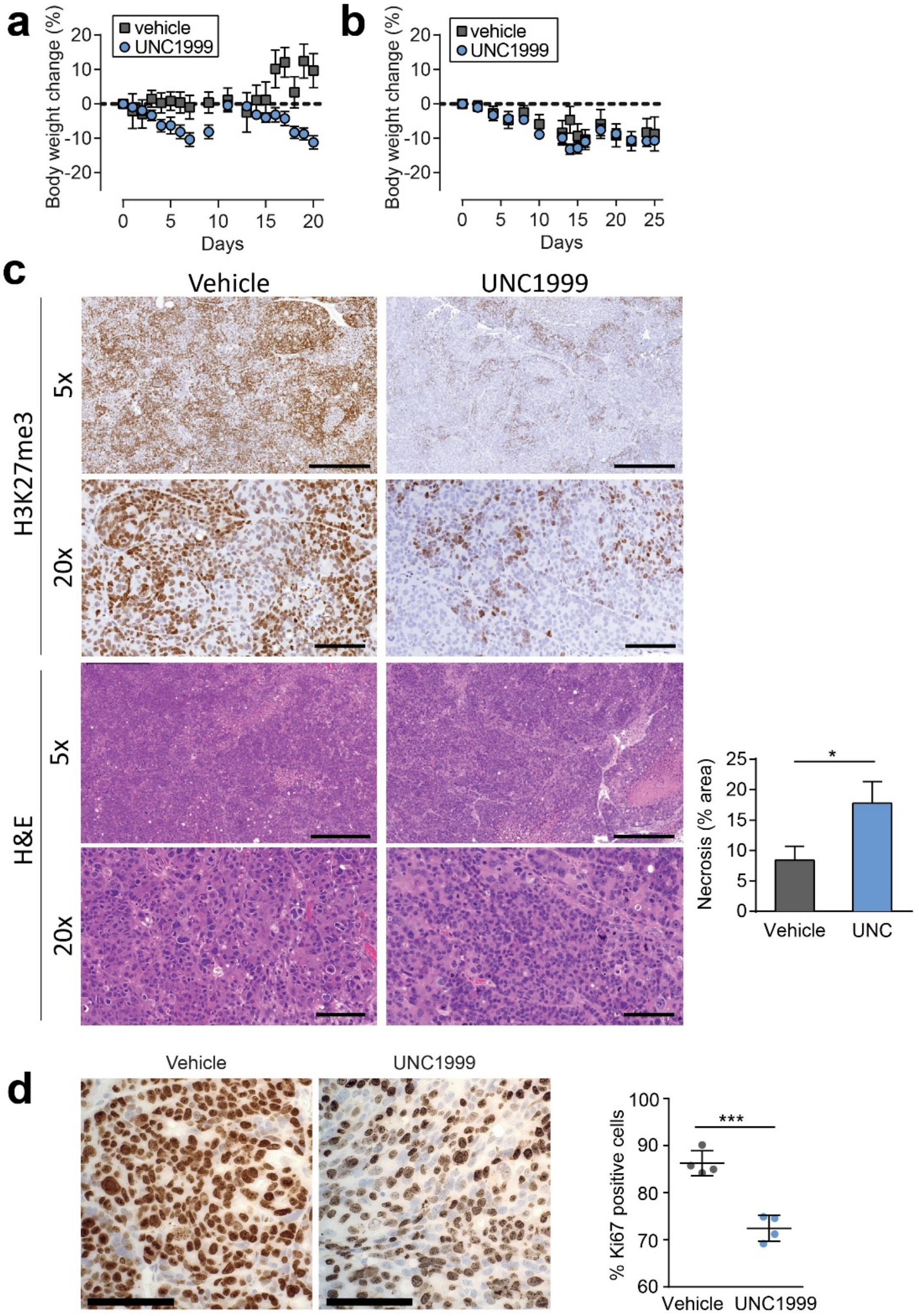

Supplementary Figure 3. Inhibition of EZH2 reduces H3K27me3 *in vivo*. (**a, b**) Body weight of SCID mice engrafted with POP92 xenografts (**a**) or POP66 xenografts (**b**) and dosed with UNC1999. Body weight decrease was calculated as a percentage of the body weight at day 0 of treatment. Data is n=10 mice (**a** and **b**-Vehicle) or n=15 mice (**b-** UNC1999) +/- SEM. (**c**) H&E & H3K27me3 IHC staining of POP92 xenografts treated with Vehicle or UNC1999. Scale bar is 500μm (5x) and 100μm (20x). Pictures shown are representative of n=6 tumours (H3K27me3) and n=14 (H&E). Histogram depicts necrosis quantification performed on the H&E stained samples. Necrosis area is calculated as a percentage of the entire surface of the tissue on the slide. Data shown are mean of n=14 tumours +/- SEM. A Student's t test was used to calculate statistical significance. (**d**) Ki67 staining of Vehicle and UNC1999 treated tumours. Images show representative areas of the tumour. Scale bar is 200μm (40x). Quantification of 5-6 randomly acquired fields per tumour, n=4 tumours per group, was performed and plotted on the right. Data are n=4, +/- SEM, Student t-test. *P<0.05, *** P<0.001.

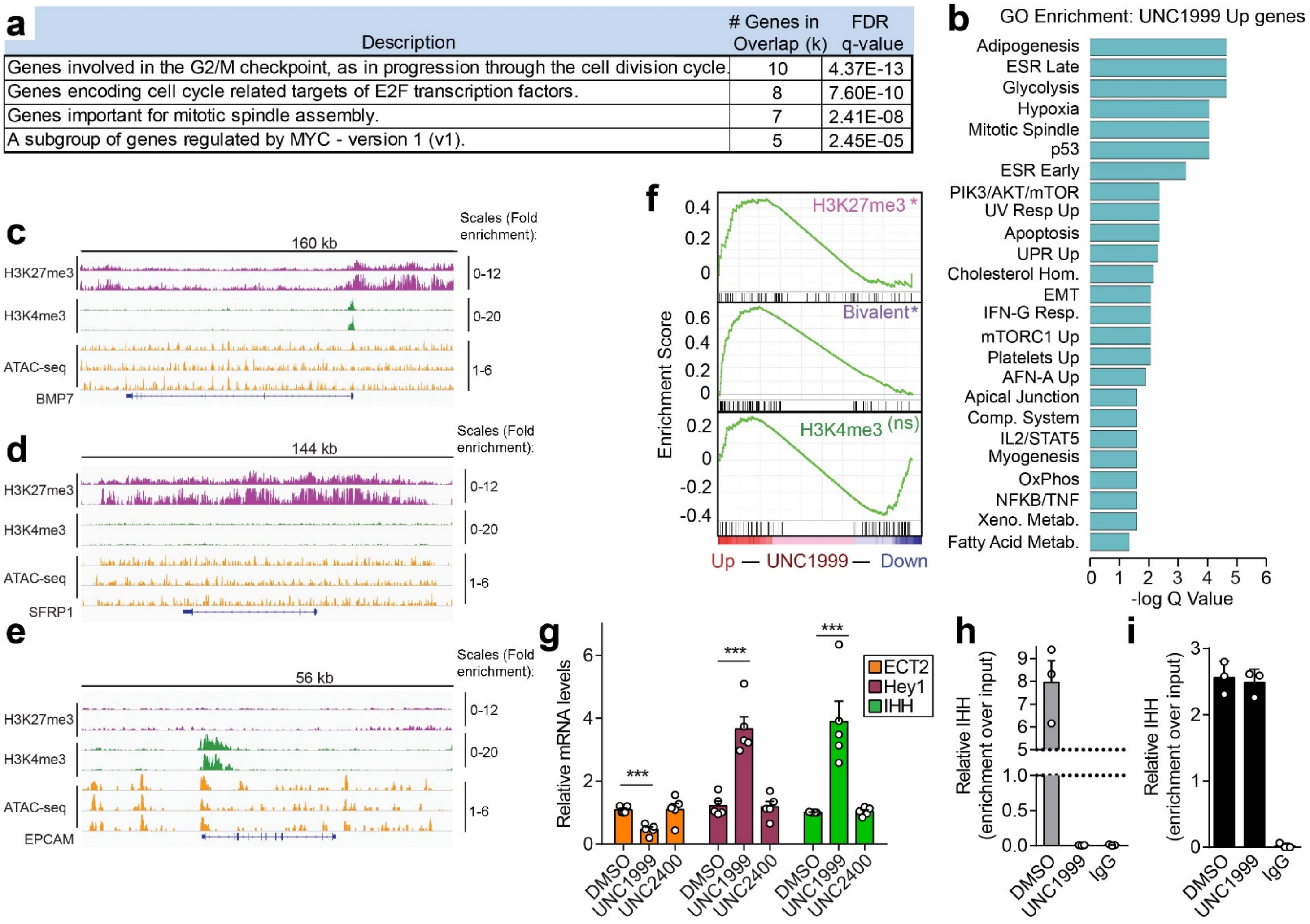

Description	# Genes in Overlap (k)	FDR q-value
Genes involved in the G2/M checkpoint, as in progression through the cell division cycle.	10	4.37E-13
Genes encoding cell cycle related targets of E2F transcription factors.	8	7.60E-10
Genes important for mitotic spindle assembly.	7	2.41E-08
A subgroup of genes regulated by MYC - version 1 (v1).	5	2.45E-05

Supplementary Figure 4. GO term analysis for UNC1999-treated POP92 and ChIP-seq tracks for H3K27me3, H3K4me3 or bivalently marked promoters. (**a-b**) GO terms for the 50 genes significantly downregulated (**a**) and for the 333 genes significantly upregulated after UNC1999 treatment (**b**) (FDR corrected, hypergeometric tests). (**c-e**) ChIP-seq and ATAC-seq tracks in POP92 spheroids for representative genes with bivalent promoters (BMP7, (**c**)), H3K27me3 repressed promoters (SFRP1, (**d**)), and H3K4me3 active promoters (EPCAM, (**e**)). (**f**) Enrichment analyses of the ChIP-seq for H3K27me3, H3K4me3, and bivalently marked promoters and RNA-seq increased/decreased genes. (permutation-based approach, see methods). (**g**) RT-qPCR control of RNA-seq upregulated genes IHH and Hey1, and RNA-seq downregulated gene ECT2. Data shown are mean of *n*=5 +/- SEM, one-way ANOVA. (**h-i**) ChIP-qPCR for H3K27me3 (**h**) and H3K4me3 mark (**i**) or IgG control, on POP92 following 3μM UNC1999 treatment, for 7 days. The level of IHH promoter was measured by qPCR and normalized to input. Data shown are mean of *n*=3 +/- SEM. * $P<0.05$, *** $P<0.001$.

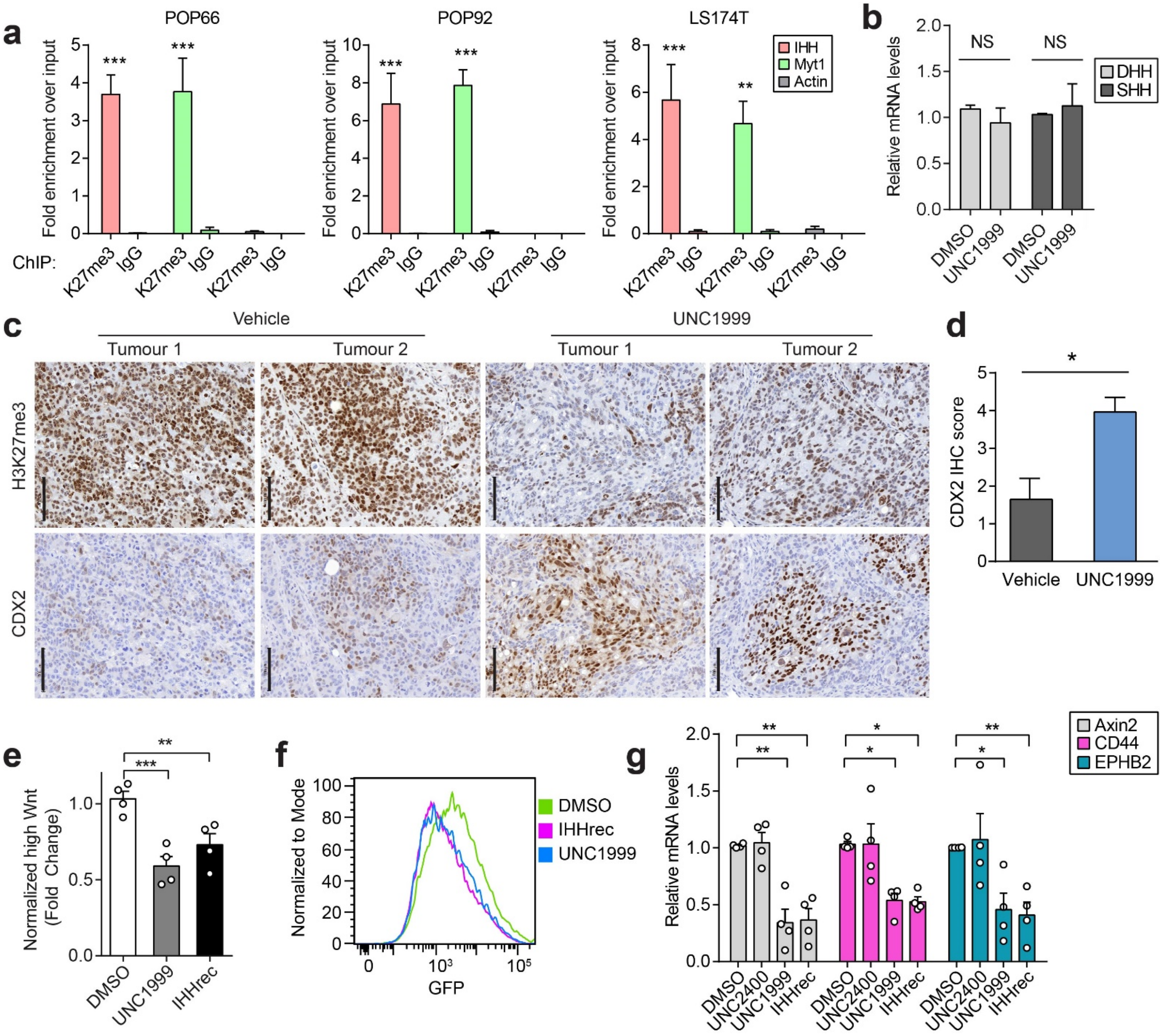

Supplementary Figure 5. EZH2 regulates CC-IC growth through control of IHH expression. (**a**) ChIP-PCR for H3K27me3 or IgG control in POP66, POP92 and LS174T. ChIP samples were processed for qPCR using primers for IHH, positive control Myt1 and negative control Actin. Data are *n*=3 +/- SEM, two-way ANOVA. (**b**) DHH and SHH mRNA levels monitored by RT-qPCR following UNC1999 treatment. Data shown are mean *n*=5 +/- SEM, two-way ANOVA. (**c-d**) IHC stainings for H3K27me3 (upper panels) and differentiation marker CDX2 (bottom panels) in Vehicle and UNC1999 treated POP92 xenografts. Representative images of xenografts from 4 different mice are shown. Scale bar is 100 µm. Magnification is 20x. Quantification of the CDX2 staining shown in (**d**). Data shown are *n*=4 +/- SEM, Student's t-test. (**e-f**) TCF/LEF-GFP Wnt reporter POP 92 cells were treated with recombinant IHH (5µg/mL) or UNC1999 (3µM) for 10 days. The data are normalized to the 10% Wnt-High cells which enriches for CC-ICs. Data in (**e**) are mean of *n*=4, +/- SEM, one-way ANOVA). Flow plot shown in (**f**) is representative for *n*=4 independent experiments. (**g**) WNT target genes Axin2, CD44, and EPHB2 monitored by RT-qPCR in POP92 cells treated with UNC2400 or UNC1999 (3µM), or recombinant IHH (5µg/mL) respectively. Data are *n*=4 +/- SEM, 2-way ANOVA. * $P<0.05$, ** $P<0.01$, *** $P<0.001$.

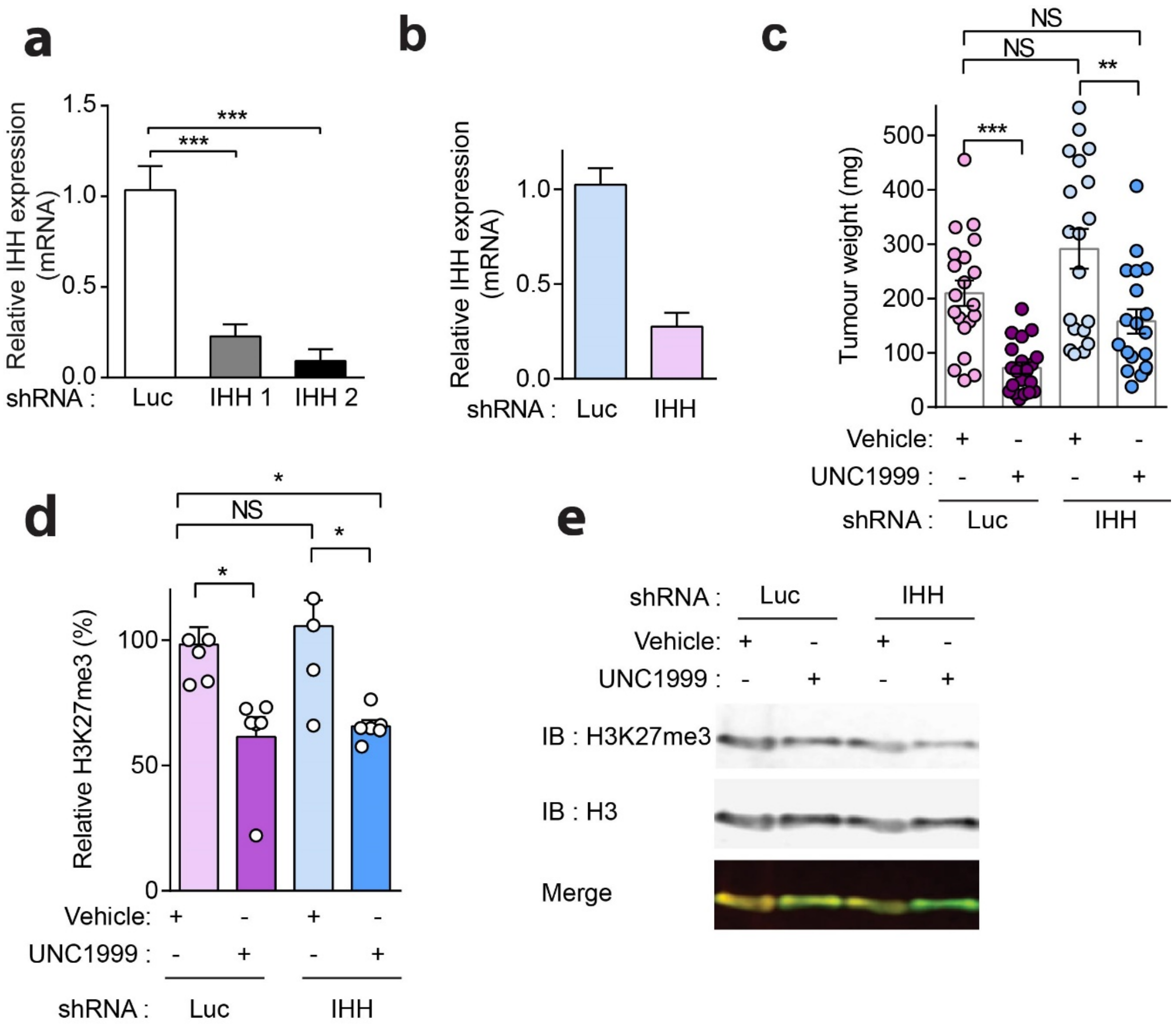

Supplementary Figure 6 : IHH knockdown in vitro and in vivo rescues the effect of UNC1999. (**a**) Validation of shRNA knockdown of IHH through RT-qPCR using IHH primers in POP92 for the viability assay in Figure 5g (**a**). Data are *n*=3 +/- SEM, one-way ANOVA. (**b-e**) POP92 spheroids infected with either shRNA against Luciferase (shLuc) or shRNA targeting IHH (shIHH) for *in vivo* experiments. Data shown in (**b**) are technical quadruplicate from POP92 IHH KD cells prior to the injection in mice for the UNC1999 rescue experiment in Figure 5i. IHH KD cells were then injected into mice and treated with Vehicle or UNC1999 (300mg/kg). Tumour weight measured upon harvesting of the xenografts (**c**). Data are *n*=20 tumours (shLuc +/- UNC1999), n=19 tumours (shIHH +/- UNC1999), +/- SEM, two-way ANOVA. Assessment of H3K27me3 levels by Western blot from xenografts harvested in Figure 5i is shown in (**d**). The quantification in (**d**) is relative to shLuc Vehicle, *n*=6, +/- SEM, two-way ANOVA. A representative blot for *n*=6 tumours is shown in (**e**). * *P*<0.05, ** *P*<0.01, ****P*<0.001.

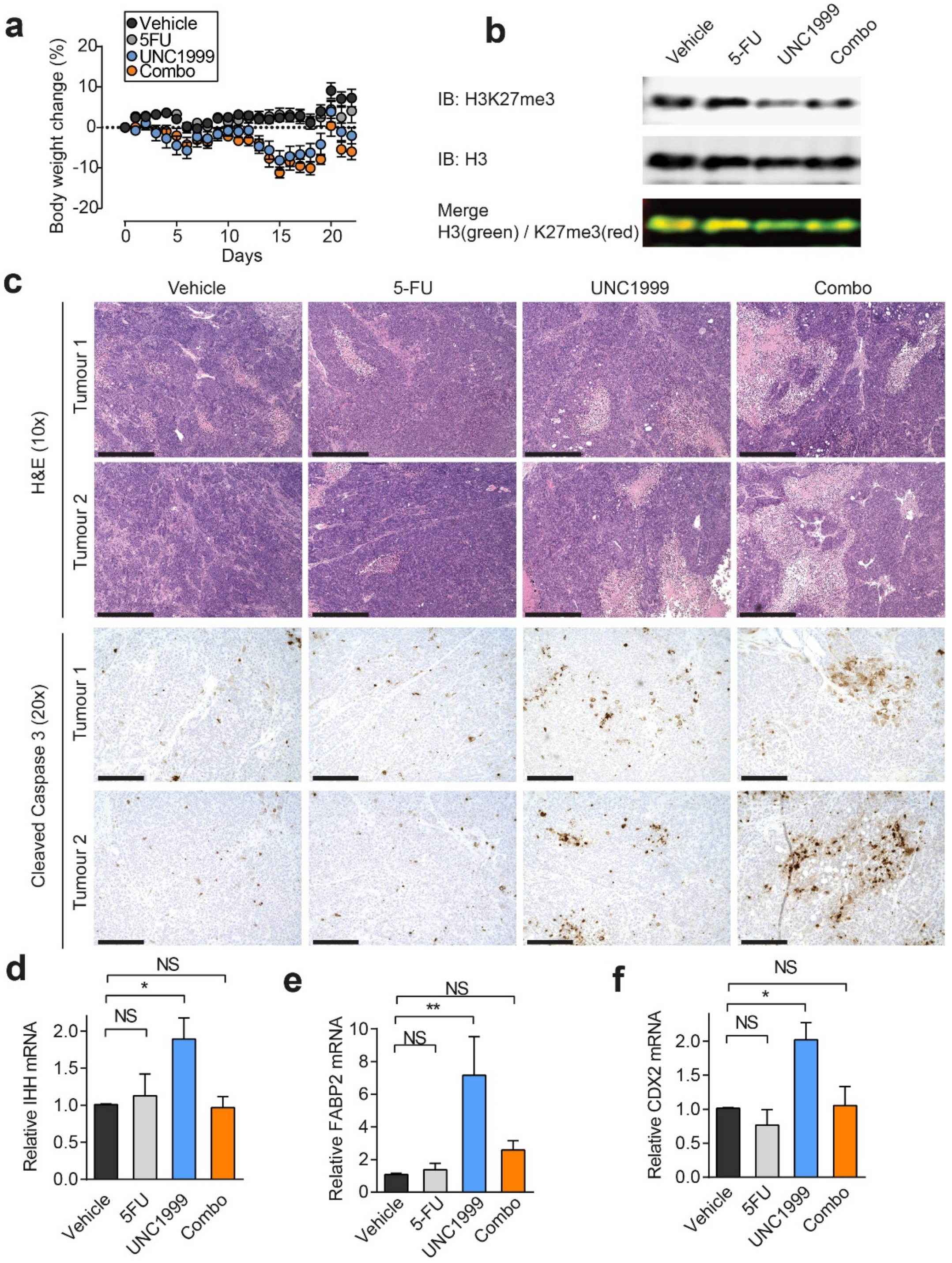

Supplementary Figure 7. **Body weight, H3K27me3 levels and expression of differentiation markers following UNC1999 treatment *in vivo* in combination with 5-FU.** (**a**) Body weight measured in SCID mice dosed as indicated. Body weight loss was calculated as a percentage of the body weight measured at day 0 of treatment. Data is *n*=7 mice (Vehicle), *n*=11 mice (UNC1999), *n*=6 mice (5-FU) or *n*=9 mice (combo) +/- SEM. (**b**) Western blot of xenograft extracts probed for decrease of H3K27me3. Blots shown are representative of *n*=7 tumours. (c) Histology analysis of xenografts generated in (**a**). H&E was performed to assess necrosis (upper panels, 10x magnification, scale bar 500µm), as well as cleaved caspase 3 for apoptosis (lower panels, 20x magnification, scale bar 200µm). (**d-f**) RT-qPCR for IHH (**d**), and the differentiation markers FABP2(**e**) and CDX2(**f**) performed on xenografts. Data shown are *n*=6 tumours (d), n=5 (e), n=4 (f) +/- SEM, one way ANOVA. *P<0.05, ** P<0.01.
