# Supplementary material for: Targeting bivalency de-represses Indian Hedgehog and inhibits self-renewal of colorectal cancer-initiating cells
Source: Nat Commun. 2019 Mar 29;10:1436. doi: 10.1038/s41467-019-09309-4 (PMC6441108; doi:10.1038/s41467-019-09309-4)
Supplement: Supplementary file 3 — Description of Additional Supplementary Files [file 41467_2019_9309_MOESM3_ESM.pdf]

Description of Additional Supplementary Information

File Name: Supplementary Data 1
Description: RNA-seq gene list of significantly upregulated genes following UNC1999 treatment.

File Name: Supplementary Data 2
Description: Cis-regulatory element analysis performed on transcriptionally accessible chromatin.
